# Computational drug discovery of potential 5α-reductase phytochemical inhibitors and hair growth promotion using *in silico* techniques

**DOI:** 10.3389/fbinf.2025.1570101

**Published:** 2025-05-06

**Authors:** Behnam Hasannejad-Asl, Farkhondeh Pooresmaeil, Sareh azadi, Ali Najafi, Ali Esmaeili, Saeid Bagheri-Mohammadi, Bahram Kazemi

**Affiliations:** ^1^ Student Research Committee, Department of Medical Biotechnology, School of Advanced Technologies in Medicine, Shahid Beheshti University of Medical Sciences, Tehran, Iran; ^2^ Department of Biotechnology, School of Advanced Technologies in Medicine, Shahid Beheshti, University of Medical Sciences, Tehran, Iran; ^3^ Department of Medical Biotechnology, School of Allied Medicine, Iran University of Medical Science, Tehran, Iran; ^4^ Department of Animal Biology, Faculty of Natural Sciences, University of Tabriz, Tabriz, Iran; ^5^ Student Research Committee, Department of Tissue Engineering and Applied Cell Sciences, School of Advanced Technologies in Medicine, Shahid Beheshti University of Medical Sciences, Tehran, Iran; ^6^ Department of Paramedicine, Amol School of Paramedical Sciences, Mazandaran University of Medical Sciences, Sari, Iran

**Keywords:** androgenetic alopecia, 5α-reductase inhibitors, herbal extracts, computational study, hair follicle growth

## Abstract

**Introduction:**

Male pattern hair loss (MPHL), also known as androgenetic alopecia (AGA), is a common disorder primarily caused by dihydrotestosterone (DHT). The Food and Drug Administration (FDA) has approved two 5-alpha reductase (5-AR) inhibitors—finasteride and dutasteride—for treating this condition. However, recent studies have reported adverse sexual side effects and issues with sperm production in young men using these medications. There are also recommendations for effectively treating hair loss with natural remedies, such as *Urtica dioica (nettle)*, *Serenoa repens (saw palmetto)*, and *Trigonella foenum-graecum (fenugreek)* that is mainly used for diminish the hair loss in the traditional medicine. Research shows that these herbal formulations and plant extracts may help reduce hair loss. However, the concentration of active compounds in these herbal extracts is often low, necessitating a large extract volume to achieve noticeable effects on hair growth. Although many studies have investigated the effects of these herbal extracts on hair growth, fewer studies focus on the specific compounds influencing the molecular mechanisms of hair loss, particularly the inhibition of 5-AR.

**Methods:**

For the first time, we aimed to applied a computational study to explore the phytochemicals extracted from these herbs to identify compounds that can effectively bind to and inhibit 5-AR. Additionally, we assessed the stability of the ligands encapsulated in lipid nanoparticles (LNP) by conducting molecular dynamics (MD) simulations of the LNP-encapsulated ligands. We utilized an online database to identify compounds from the extracts of nettle, saw palmetto, and fenugreek. We then analyzed their binding affinity to 5-AR using computational techniques.

**Results:**

We found that 6 molecules—Jamogenin, Neodiosgenin, Chlorogenic acid, Rutin, Riboflavin, and Ursolic acid—are effective in binding to 5-AR. Additionally, our *in silico* studies revealed that vesicle-entrapped JAMOGENIN, which has a stronger bond with 5-AR, is more stable than its unencapsulated form.

**Discussion:**

Therefore, these 6 molecules, particularly JAMOGENIN, should be considered for experimental analysis in both their unencapsulated and nanocarrier-encapsulated states to promote hair follicle growth.

## 1 Introduction

The interactions between androgens and androgen receptors can significantly affect cellular processes that regulate key functions related to prostate development, structure, and activity as well as male pattern baldness. Additionally, androgens may contribute to the development of prostate cancer. Several preclinical and clinical studies have indicated that suppressing dihydrotestosterone (DHT) may help prevent the onset of cancer (Lieberman, 2003).

The membrane-embedded enzyme human steroid 5α-reductase (5-AR) plays a vital role in converting the primary androgen testosterone to its more potent form DHT. Both testosterone and DHT can bind to androgen receptors, but DHT has a significantly higher affinity (approximately two to five times greater) than testosterone. Moreover, DHT is approximately 10 times more potent than testosterone. As a result, signals mediated by DHT are primarily facilitated through the androgen receptors (Ali et al., 2024). DHT is essential for the proper development of male external genitalia and the prostate; it also influences various human diseases, including male pattern hair loss (MPHL), hirsutism, acne, benign prostatic hyperplasia (BPH), prostate cancer, and androgenetic alopecia (AGA) (Chakraborty et al., 2023).

MPHL is the gradual loss of scalp hair that affects both men and women; it is primarily caused by hormonal factors and genetics and is a common chronic skin condition with global incidence (Lolli et al., 2017; Abbas et al., 2021). By the age of 70 years, MPHL affects at least 80% of men and approximately 50% of women, and its prevalence increases with age. This condition poses considerable challenges in management, especially in terms of choosing the right treatment options (Nestor et al., 2021). DHT and 5-AR are the primary mediators of this disorder, where DHT interacts with the androgen receptors in vulnerable scalp hair follicles to stimulate the genes responsible for follicular miniaturization (Iltaf et al., 2021). The commonly known United States Food and Drug Administration (FDA)-approved treatments for hair loss include topical minoxidil, oral finasteride, and low-level light therapy. Minoxidil was originally developed as an antihypertensive medication owing to its ability to dilate blood vessels; however, it was unexpectedly found to promote hair growth. When applied to the scalp, minoxidil improves blood circulation and increases vascularity in the area. Finasteride consumed orally works by blocking 5-AR; although it was initially developed to treat the symptoms of BPH, it is also used to treat AGA in men (Choi et al., 2024; Dhariwala and Ravikumar, 2019). The combination therapy of finasteride and minoxidil can significantly improve hair health and is generally more effective than either treatment alone (Leavitt et al., 2005); however, both treatments may have adverse effects over time (Nestor et al., 2021). Finasteride can lead to scalp itching, burning, irritation, contact dermatitis, redness, increased liver enzymes, nighttime urination issues, and sexual dysfunction, including impotence (Devjani et al., 2023). Conversely, minoxidil may result in irritant and allergic contact dermatitis, headaches, and excess facial hair growth (Nestor et al., 2021). Therefore, it is essential to recognize natural components that can serve as safer choices for treating hair loss. Accordingly, one favorable area of research is the examination of plants that demonstrate 5-AR inhibitor activities (Korassa et al., 2022).

The growing popularity of plant-based medications can be attributed to their established safety profiles in recent years (Srivilai et al., 2019). Phytochemicals are a class of metabolites produced by plants to enhance their survival strategies and are not found in mammals. Depending on their chemical structures, phytochemicals can be broadly categorized into phenolic compounds, terpenes/terpenoids, nitrogen-containing compounds, and sulfur-containing compounds. These compounds exhibit wide ranges of physicochemical, biochemical, and biological functions (Kaur et al., 2022; Al-Khayri et al., 2023). Many phytochemicals have been identified as stimulants for hair growth, including curcumin, garlic gel, red ginseng extract, capsaicin, procyanidin, pumpkin seed oil, rosemary oil, and saw palmetto (Kesika et al., 2023). Integrating various phytochemicals from different botanical sources allows formulation of effective herbal or natural cosmetic products that can promote hair growth (Brandt et al., 2011). Research into phytochemicals is necessary to evaluate the therapeutic properties of a plant and identify the active compounds responsible for its biological effects. The key phytochemical components found in plant extracts, such as phenolic compounds, terpenes, terpenoids, sulfur-containing compounds, fatty acids, and others, are associated with their biological activities that support hair growth.

Current experimental evidence is inadequate for proving associations between structures and activities, implying that further investigations are required to improve drug development for hair loss (Choi et al., 2024). Moreover, filtering bioactive compounds for their pharmacological efficacies is often time-consuming, resource-intensive, and expensive. In the twenty-first century, computational methods present a promising innovative approach, particularly as they can be used to focus on medicinal plants to develop new drugs. Techniques such as molecular docking, molecular dynamics (MD) simulations, and artificial intelligence play crucial roles during *in silico* research on medicinal plants. The small-molecular docking method is used to analyze how potential phytochemicals interact with their target binding pockets, and this provides insights into their interactions and therapeutic results. MD simulations reveal the dynamic behaviors of biomolecules at the atomic level, offering detailed representations of the biomolecular dynamics (Singh and Bharadvaja, 2021).

Recent studies indicate that natural extracts and compositions derived from plants, such as *Urtica dioica* (nettle), *Serenoa repens* (saw palmetto), and *Trigonella foenum-graecum* (fenugreek), may help reduce hair loss both directly and indirectly (Gasmi et al., 2023; Singh et al., 2022). *U. dioica* is a promising herbal candidate for promoting hair health and has been traditionally used to address various non-scarring types of alopecia. Some encouraging preclinical studies have also supported its therapeutic effects. Its antioxidant properties may help protect hair from damage caused by free radicals—a primary factor in hair loss and damage (Ivanivna et al., 2018; Pekmezci and Türkoğlu, 2023). Another important plant is *S. repens*, whose extracts or oils contain high concentrations of fatty acids (85%–90%) and exhibit anti-inflammatory, anti-androgenic, and anti-proliferative properties. *S. repens* is recognized as a natural 5-AR inhibitor and has gained popularity as a potential treatment for AGA. Its functions are similar to those of finasteride for inhibiting the enzyme 5-AR. However, research has shown that liposterolic extracts of *S. repens* (LSESr) are more effective at inhibiting 5-AR *in vitro* than finasteride. Therefore, using natural remedies like *S. repens* may help reduce the adverse effects of finasteride (Dhariwala and Ravikumar, 2019; Rondanelli et al., 2016; Zhu et al., 2018).

*T. foenum-graecum* is a traditional herbal medicine known for stimulating hair growth; this plant contains several active compounds, including saponins, flavonoids, alkaloids, and steroids. Additionally, it is rich in fiber, proteins, linoleic and linolenic acids, as well as vitamins A, B1, B2, and C (Tewari et al., 2024). Research has shown that its oil can effectively prevent hair loss and baldness owing to its high protein and nutritional content, which nourishes the hair and scalp. Furthermore, it helps prevent lice and dandruff as well as adds strength and flexibility to dry, damaged, and brittle hair to promote overall hair growth (Wasiullah et al., 2022).

Previous hair growth methods, especially those entailing FDA-approved 5-AR inhibitors like finasteride and dutasteride, have been linked to significant adverse side effects, including sexual dysfunction and problems with sperm production in younger men. On the other hand, the concentrations of active compounds in natural remedies such as *U. dioica*, *S. repens*, and *T. foenum-graecum,* which are often recommended for hair loss treatment, tend to be low; this requires the use of large volumes of herbal extracts to achieve noticeable results. Additionally, many studies are focused on evaluating the overall effects of herbal extracts on hair growth rather than on specific compounds responsible for influencing the molecular mechanisms related to hair loss, particularly the inhibition of 5-AR. Traditional methods often utilize only limited computational techniques that could offer insights into molecular interactions and stability. These limitations underscore the need for more effective and safer solutions for promoting hair growth. The present study aims to identify potential compounds derived from *U. dioica*, *S. repens*, and *T. foenum-graecum* that have therapeutic properties. We investigated the manner in which these selected candidates interacted with 5-AR through small-molecule docking and MD simulations. Our findings showed that six phytochemicals present in these plant extracts could effectively bind to the 5-AR binding pocket. Among these, jamogenin was the most promising 5-AR inhibitor. We encapsulated jamogenin in lipid nanoparticles (LNPs) and simulated it in a dynamic environment using MD simulation, which showed that it was more stable in the encapsulated condition. Thus, further studies are warranted on these jamogenin LNPs to evaluate its 5-AR inhibition activity and potential effects on hair growth.

## 2 Materials and methods

### 2.1 List of herbal compounds

Dr. Duke’s Phytochemical and Ethnobotanical Databases (https://phytochem.nal.usda.gov/) were utilized to identify and compile a list of compounds from our selected medicinal herbs: *S. repens*, *T. foenum-graecum*, and *U. dioica* (Duke, 2023). This database allows comprehensive searches of plants, chemicals, bioactivities, and ethnobotany using common or scientific names, which appeal to researchers concentrating on herbal products, alternative medicines, nutrition, and biomedicine. Additionally, users can download their search results in spreadsheet or PDF format.

### 2.2 Capturing or predicting the three-dimensional structures and preprocessing the compounds

The structures of the list of compounds were downloaded in structured data file format from the PubChem database (https://pubchem.ncbi.nlm.nih.gov/) (Kim et al., 2023). We obtained the canonical International Union of Pure and Applied Chemistry (IUPAC) names for the compounds whose structures were unavailable on PubChem. We then used the ChemDraw Professional 17.0 tool from the ChemOffice package (Zielesny, 2005) to draw the structures of these compounds. Next, we utilized the Chem3D tool from ChemOffice (Zielesny, 2005) to carry out energy minimization in two steps; here, the mm2 and MD modules were applied, and the minimized structures were saved as protein data bank (PDB) files. Furthermore, we employed the graphical interface Chimera version 1.17.1 tool (Pettersen et al., 2004) to add the Gasteiger charges and merge the non-polar hydrogen atoms to prepare the energy-minimized molecules for docking. Finally, we detected the aromatic carbon rings to establish a torsion tree, and the prepared molecules were saved as PDBQT files.

### 2.3 Capturing the protein structure and preparing for molecular docking

The crystal structure of the steroid 5-AR complexed with finasteride at a resolution of 2.80 Å (PDB ID: 7BW1) was obtained from the RCSB PDB (https://www.rcsb.org/) (Burley et al., 2023). The graphical interface Chimera version 1.17.1 (Pettersen et al., 2004) was then used to remove the complexed crystal ligands (finasteride), water molecules, and ionic metals from the structure. Subsequently, Gasteiger charges were added, and non-polar hydrogen atoms were merged. Additionally, the aromatic carbon rings were identified to prepare the ligands for establishing a torsion tree. Finally, the resulting molecule was minimized for energy using the AMBER force field over 1,000 steps and saved as a PDBQT file.

### 2.4 Molecular docking

AutoDock Vina version 1.12.1 (Trott and Olson, 2010) is an open-source tool used for ligand–receptor docking. We utilized PyRx version 0.9.2 (Dallakyan and Olson, 2015) as the interface for docking purposes as it is integrated with AutoDock Vina. First, the prepared protein was input as a macromolecule, and the prepared small molecules were introduced as ligands to the system. Subsequently, a grid box was centered at −24.3258265956X, 14.5429831684Y, and 31.4381014102Z on the surface of 5-AR with a grid point spacing of 0.375 Å, which encompassed the binding site residues, to perform the docking.

### 2.5 Pharmacokinetic analysis

The top complexes identified from the ligand–receptor docking results were selected based on their numbers of hydrogen bonds with the binding site residues and favorable free Gibbs binding energy (ΔG) values. We then examined their pharmacokinetic properties using the SwissADME web server (http://www.swissadme.ch/) (Daina et al., 2017). This platform allows us to predict the pharmacokinetic characteristics; medicinal chemistry suitability; drug-like properties; absorption, distribution, metabolism, excretion, and pharmacokinetics (ADME) parameters; and physical characteristics of small molecules for drug design purposes.

### 2.6 Ligand–receptor MD simulations

The final ligand–receptor complexes selected for further analyses were subjected to MD simulations. First, the ligand–receptor complexes were separated, and the ligand topology was created using the CHARMM General Force Field (CGenFF) web server based on the charmm36 force field (https://app.cgenff.com/login) (Lee et al., 2016). This force field was chosen because it is accurate and robust for simulating biomolecular interactions. Additionally, the core modules of GROMACS version 2023 (Abraham et al., 2023) were employed to construct the receptor topology using the same force field. Applying the same force field parameters to both ligands and receptors ensures consistency and reliability. Next, GROMACS version 2023 was used as an open-source tool to perform the MD simulations. Each simulation system was configured by placing the complex at the center of a dodecahedral simulation box exhibiting periodic boundary conditions (PBCs) to minimize the edge effects. The system was then solvated in a three-site transferable water model (TIP3P) to mimic the aqueous environment. Na^+^ and Cl^−^ ions were next added to neutralize the system to achieve a concentration of 0.15 M to reflect physiological conditions. A 5000-step minimization was then performed using the steepest descent algorithm after neutralization to remove any steric clashes or unfavorable interactions. This was followed by equilibration of the system over 3 ns and 5 ns in the constant number of particles, volume, and temperature (NVT) and constant number of particles, pressure, and temperature (NPT) ensembles, respectively. During this optimization, the Nose–Hoover thermostat (Evans and Holian, 1985) was employed for its effectiveness in maintaining temperature stability. The Nose–Hoover thermostat is a widely used method in MD simulations as it creates a canonical ensemble by coupling the system to an external heat bath. This method allows precise temperature variation regulation to guarantee that the system stays at the ideal temperature throughout the simulation. Because of its capacity to produce appropriate equilibrium distributions, it is a common option for simulations needing precise temperature adjustments (Evans and Holian, 1985). Meanwhile, the Parrinello–Rahman barostat (Parrinello and Rahman, 1981) was used to control the pressure to offer precise pressure regulation for the system. The Parrinello–Rahman barostat is particularly suitable for complex system simulations as it allows anisotropic scaling of the simulation box. This implies that the shape and size of the simulation box can be independently changed in different directions, which is crucial for maintaining proper pressure and density of the system. The Parrinello–Rahman barostat enables the creation of a stable and realistic simulation box for ensuring that the system stays at the desired pressure of 1 bar (Parrinello and Rahman, 1981). The cutoff value for the van der Waals (vdw) and electrostatic interactions was set to 12 Å to ensure accurate interaction calculations within this range, and the particle mesh Ewald (PME) method was used for long-range electrostatic calculations to obtain precise and efficient computations of the electrostatic interactions. The temperature was maintained at 310 K using the Nose–Hoover thermostat with a time constant of 0.2 ps. At the same time, the pressure was maintained at 1 bar with the Parrinello–Rahman barostat, which was also set to a constant time of 0.2 ps. The choice of these thermostat and barostat enhances the stability and accuracy of the simulations. To preserve constant hydrogen bonding and guarantee the structural integrity of the system throughout the simulations, the linear constraint solver (LINCS) method (Hess et al., 1997) was used; this is a popular algorithm for handling bond restrictions in MD simulations because it resets the restrictions instead of their derivatives while helping to avoid drift, thereby ensuring intrinsic stability. This stability is essential for accurate and dependable simulation findings (Hess et al., 1997). Thus, the MD simulations were conducted for 50 ns by recording the coordinates and energies of the trajectories every 10 ps to yield an extensive and thorough dataset for the study. The internal modules of GROMACS were used to analyze the MD simulation trajectory results. To assess the stability of the system, we analyzed the root mean-squared fluctuation (RMSF), root mean-squared deviation (RMSD), and radius of gyration (Rg).

### 2.7 MD simulations of vesicle- and polymer-enveloped ligands

We designed an envelope structure containing bilayer vesicles to evaluate the stabilities of select ligands under various encapsulation states within LNPs against free (unencapsulated) ligands. The CHARMM-GUI web server (Jo et al., 2008) was used to prepare the structures of these carriers. The bilayer vesicle was created using a coarse-grained (CG) model based on the martini22 force field (De Jong et al., 2013) and was subsequently equilibrated through six cycles of NVT and NPT simulations. Thereafter, the CG model of the vesicle was converted to an all-atom (AA) model using the charmm36 force field. TIP3P water molecules were then added to create a simulation box for both the free and vesicle-encapsulated ligands. The systems were then neutralized by adding 0.15 M of Na^+^ and Cl^−^ ions. Finally, the energies of both systems were minimized and equilibrated according to the parameters outlined earlier, followed by MD simulations lasting 20 ns.

## 3 Results

### 3.1 List of herbal compounds

Dr. Duke’s Phytochemical and Ethnobotanical databases (Duke, 2023) containing data on plant chemicals were used to extract the chemical compositions of *S. repens*, *T. foenum-graecum*, and *U. dioica*. We searched for these plants individually and downloaded the results as Excel files in June 2023. Thus, *S. repens*, *T. foenum-graecum*, and *U. dioica* were documented to have 129, 351, and 320 chemical compounds, respectively (Supplementary Table S1). After removing duplicate substances and metals such as zinc, the final list for *S. repens* included 38 unique compounds while *T. foenum-graecum* and *U. dioica* had 127 selected compounds each, which were used for further analyses (Supplementary Table S2). The IUPAC names and canonical SMILES data of the chosen small molecules were obtained from the PubChem database and used to predict the 3D structures of compounds that did not have entries in the database.

### 3.2 Molecular docking

**FIGURE 1:**
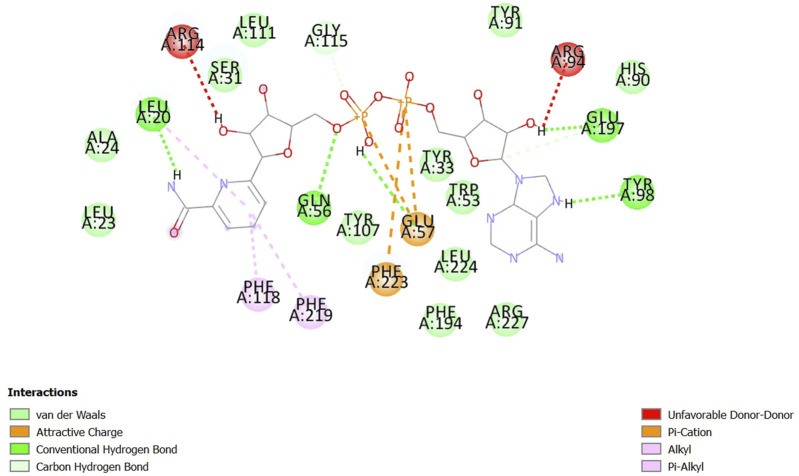
Schematic interaction of fenugreekine with 5-AR. The docked fenugreekine shows five conventional hydrogen bonds, two carbon and hydrogen bonds, three pi-cation bonds, and three pi-alkyl bonds with the binding pocket residues of 5-AR.

The open-source tool AutoDock Vina integrated with PyRx was used to assess the binding affinities of ligands to a selected receptor and analyze the interactions thereof. A total of 21 candidates from *S. repens*, 36 from *T. foenum-graecum*, and 47 from *U. dioica* were identified, where each formed at least one hydrogen bond with the residues at the preferred binding sites (Supplementary Table S3). Among all the docked ligands, 1, 11, and 7 molecules from *S. repens, T. foenum-graecum*, and *U. dioica*, respectively, exhibited three or more hydrogen bonds with the favorite residues (refer to Table 1). Notably, fenugreekine from *T. foenum-graecum* demonstrated the highest number of hydrogen bonds of five, as visualized using BIOVIA Discovery Studio Visualizer v16.1.0.15350 (Pawar and Rohane, 2021) in Figure 1. Although the formation of hydrogen bonds with the binding site residues is crucial, the binding affinities of the ligands to the receptors are another critical factor that must be considered. Molecular docking was performed three times to evaluate the binding affinities of the compounds, and the mean ΔG values from these three runs were used to represent the binding affinities of the molecules. The results indicated that the mean ΔG ranges for the candidates from *S. repens*, *T. foenum-graecum*, and *U. dioica* were −11.60 to −4.63, −12.03 to −2.00, and −11.70 to −2.60 kcal/mol, respectively (Supplementary Table S3). Ultimately, based on the abundance of favorable hydrogen bonds and mean ΔG values, we selected 16 candidates for further analyses after removing common molecules between the studied plants (one from *S. repens*, eight from *T. foenum-graecum*, and seven from *U. dioica*; see Table 1).

**TABLE 1:** List of the compounds with more than 3 favorite hydrogen bonds.

Chemical name	Plant	Mean of ΔG (KJ/mol)	Favorite Hydrogen bonds	Other Hydrogen bonds
RIBOFLAVIN	*Serenoa repens*	−10.9	3	7
CHLOROGENIC-ACID	*Urterica dioica*	−9.1	3	7
ISORHAMNETIN-3-O-RUTINOSIDE	*Urterica dioica*	−9.9	3	6
KAEMPFEROL-3-O-BETA-D-RUTINOSIDE	*Urterica dioica*	−9.03	3	5
OLEANOLIC-ACID	*Urterica dioica*	−9.3	3	0
STACHYOSE	*Urterica dioica*	−8.87	4	4
RAFFINOSE	*Urterica dioica*	−8.37	3	5
URSOLIC-ACID	*Urterica dioica*	−11.1	3	0
FENUGREEKINE	*Trigonella foenum-graecum*	−10.53	5	1
QUERCITRIN	*Trigonella foenum-graecum*	−8.8	3	5
YAMOGENIN	*Trigonella foenum-graecum*	−10	3	0
KAEMPFEROL-3,7-DIGLUCOSIDE	*Trigonella foenum-graecum*	−9.57	3	0
DIOSGENIN	*Trigonella foenum-graecum*	−10.2	4	0
QUERCETIN-3,7-DIGLUCOSIDE	*Trigonella foenum-graecum*	−9.77	4	1
RUTIN	*Trigonella foenum-graecum*	−9.7	3	7
VITEXIN-7-GLUCOSIDE	*Trigonella foenum-graecum*	−8.3	4	4

### 3.3 Pharmacokinetic properties

The SwissADME website was used to predict the pharmacokinetics and ADME characteristics of the 16 selected molecules (see Table 2). The results indicate that jamogenin and neodiosgenin can cross the blood–brain barrier (BBB) and exhibit high gastrointestinal (GI) absorption (refer to Table 2). According to Lipinski’s rule of five, which is also known as Pfizer’s rule of five or simply the rule of five (RO5) (Karami et al., 2022), the following compounds demonstrated drug-likeness: riboflavin from *S. repens*; chlorogenic acid, ursolic acid, and oleanolic acid from *U. dioica*; jamogenin and diosgenin from *T. foenum-graecum*. These molecules were selected for further investigations via MD simulations. The detailed properties of all compounds are provided in Supplementary Table S4.

**TABLE 2:** Druglikeness prediction of candidates using SwissADME website.

Plant	Chemical name	SwissADME analysis		
		MW	BBB permeant	Lipinski #violations
*Serenoa repens*	RIBOFLAVIN	376.36	No	0
*Urterica dioica*	CHLOROGENIC-ACID	354.31	No	1
*Urterica dioica*	ISORHAMNETIN-3-O-RUTINOSIDE	624.54	No	3
*Urterica dioica*	KAEMPFEROL-3-O-BETA-D-RUTINOSIDE	594.52	No	3
*Urterica dioica*	OLEANOLIC-ACID	456.7	No	1
*Urterica dioica*	STACHYOSE	666.58	No	3
*Urterica dioica*	RAFFINOSE			
*Urterica dioica*	URSOLIC-ACID	456.7	No	1
*Trigonella foenum-graecum*	FENUGREEKINE	663.43	No	3
*Trigonella foenum-graecum*	ISOQUERCITRIN	464.38	No	2
*Trigonella foenum-graecum*	YAMOGENIN	414.62	Yes	1
*Trigonella foenum-graecum*	KAEMPFEROL-3,7-DIGLUCOSIDE	610.52	No	3
*Trigonella foenum-graecum*	DIOSGENIN	414.62	Yes	1
*Trigonella foenum-graecum*	QUERCETIN-3,7-DIGLUCOSIDE	626.52	No	3
*Trigonella foenum-graecum*	RAFFINOSE	504.44	No	3
*Trigonella foenum-graecum*	RUTIN	610.52	No	3
*Trigonella foenum-graecum*	VITEXIN-7-GLUCOSIDE	594.52	No	3

Abbreviations: BBB, Blood-Brain Barrier.

### 3.4 Ligand–receptor MD simulations

**FIGURE 2:**
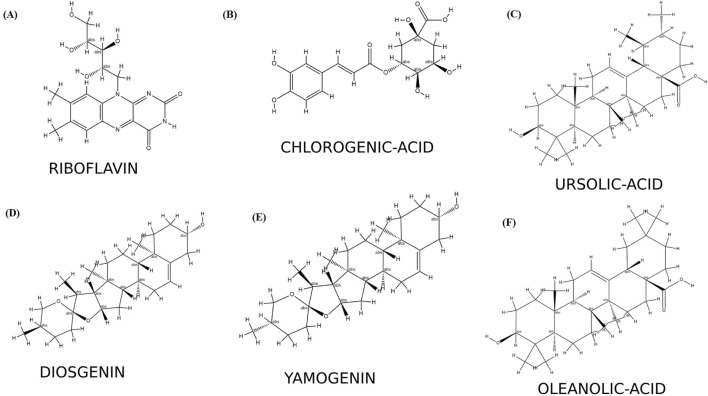
Secondary structures of the identified compounds: **(A)** riboflavin; **(B)** chlorogenic acid; **(C)** ursolic acid; **(D)** neodiosgenin; **(E)** jamogenin; **(F)** oleanolic acid.

**FIGURE 3:**
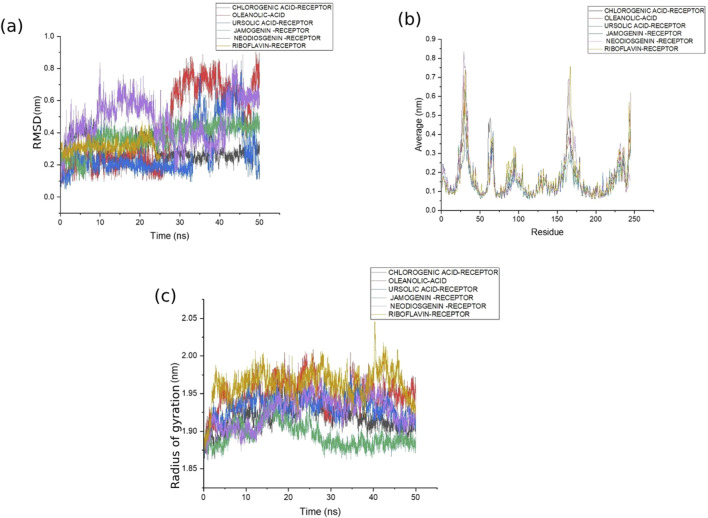
Stability assessments of the ligands with 5-AR showing the **(a)** root mean-squared deviation (RMSD), **(b)** root mean-squared fluctuation (RMSF), and **(c)** radius of gyration (Rg) of the complex structures during 50 ns of molecular dynamics (MD) simulations. In all graphs, the complexes of 5-AR with chlorogenic acid, oleanolic acid, ursolic acid, jamogenin, neodiosgenin, and riboflavin are shown in gray, red, blue, green, purple, and mustard colors, respectively.

The six selected ligands (Figure 2) exhibiting suitable binding affinities, acceptable numbers of hydrogen bonds, and favorable pharmacokinetic properties were dynamically simulated with the receptors for 50 ns. Based on assessment of the ligand–receptor complex deviations, the jamogenin-complexed receptor showed less deviation than the other ligands (Figure 3a). Specifically, this ligand experienced a 0.1 nm deviation at 7 ns and subsequently achieved a stable structure. In contrast, the neodiosgenin- and oleanolic-acid-complexed receptors exhibited the greatest deviations, with their structures fluctuating significantly throughout the simulation duration. Furthermore, the receptors complexed with ursolic acid, chlorogenic acid, and riboflavin showed deviations greater than that of jamogenin but lower than those of neodiosgenin and oleanolic acid.

Analysis of the receptor residues during MD simulations with the various ligands indicated that residues 31, 32, 30, 165, 164, and 245 displayed the most fluctuations (Figure 3b). However, the fluctuations of these residues in the complexes with ursolic acid and jamogenin were lower than those with the other complexes. In addition to the deviations and fluctuations, Rg analysis revealed that the jamogenin–receptor complex was more stable than the others (Figure 3c). Thus, based on these results of the MD simulations, jamogenin was selected for further studies.

### 3.5 Comparing the stabilities of the free and encapsulated ligands by MD simulations

**FIGURE 4:**
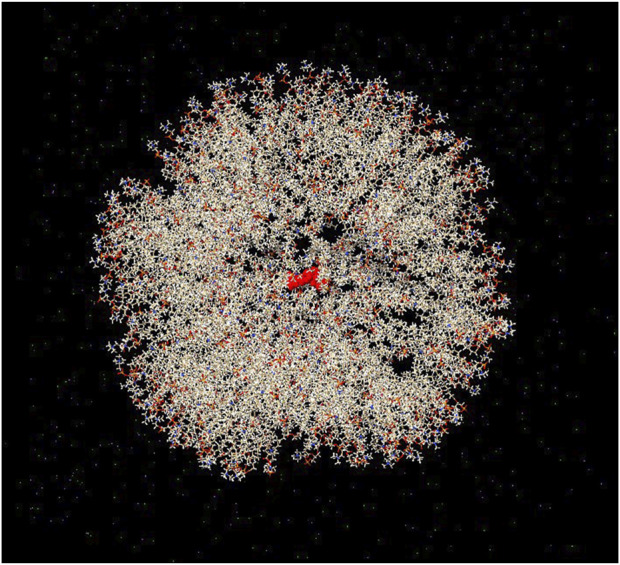
Schematic illustration of the structure of encapsulated jamogenin. The jamogenin molecule is located at the center of the bilayer vesicle, and the systemic environment contains ion molecules. The jamogenin and vesicle bilayer components are shown in red cartoon and milk-colored stick styles, respectively.

**FIGURE 5:**
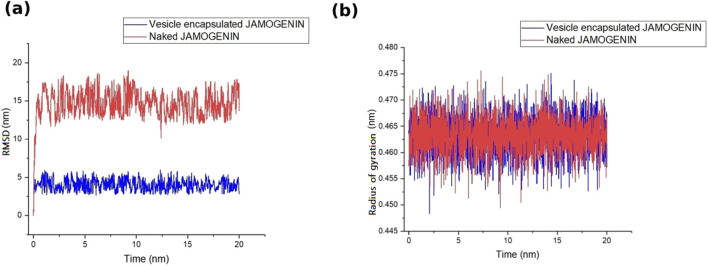
Stability assessments of the vesicle-enveloped and free (naked) jamogenin based on **(a)** RMSD and **(b)** Rg values during 20 ns of MD simulations. The vesicle-enveloped and free jamogenin are shown in blue and red colors, respectively.

In addition to drug discovery, drug delivery methods are an essential area of research. LNPs are among the most suitable carriers for transferring therapeutic cargo across cellular bilayer membranes while ensuring stability of the cargo. Bilayer vesicles, such as liposomes, are also appropriate candidates for drug delivery. In the present study, we simulated a bilayer vesicle constructed using 1-palmitoyl-2-oleoyl-glycero-3-phosphocholine (POPC) and encapsulated the jamogenin molecule at the center of the vesicle (Figure 4). This system, containing TIP3P water and neutralized ions, underwent MD simulations for 20 ns, and the results were compared to those of the free jamogenin molecule. The RMSD analysis indicates that the encapsulated jamogenin is more stable than the free molecule (Figure 5a). The unencapsulated ligand exhibited structural instability with deviations exceeding 15 nm during the initial simulation steps, whereas the encapsulated jamogenin showed lower structural instability with deviations of less than 5 nm. Furthermore, throughout the 20 ns of MD simulations, the free ligand demonstrated more significant deviations than the encapsulated jamogenin.

We also analyzed the Rg for the two systems along with the RMSD. The Rg values of both systems were similar; however, the LPN-encapsulated system demonstrated greater stability than the free ligand system (Figure 5b).

## 4 Discussion

MPHL is one of the associated consequences of excess DHT, which binds to the androgen receptors and diminishes the activities of hair growth factors. These interactions cause gradual shrinkage of the hair follicles and ultimately result in hair loss (Adil and Godwin, 2017; Abdurrahman et al., 2023). One strategy for mitigating the effects of androgens like DHT on AGA is to hinder the binding between the androgens and their receptors. The two FDA-approved medications for treating AGA, namely, finasteride and minoxidil, have limited efficacy and can cause diverse side effects. Thus, it is crucial to explore novel treatments for AGA (Kasmawati et al., 2022). Medicinal herbs are favorable candidates for discovering new compounds to treat this condition. Several studies have shown that specific herbal extracts can effectively stimulate hair growth. For example, *T. foenum-graecum*, *U. dioica*, and *S. repens* are traditional herbs commonly used to combat AGA (Dey et al., 2013; Bansal et al., 2012; González et al., 2010; Murata et al., 2013; Rossi et al., 2012). An alopecia mouse model was used to demonstrate that ethanolic extracts of *T. foenum-graecum* leaves had significantly stronger hair-growth-promoting effects than standard minoxidil (Imtiaz et al., 2017); these extracts contain beneficial compounds such as polyphenols, flavonoids, polysaccharides, and glycosaponins. When this extract was applied topically for 21 d, the hair length and diameter of the treated animals had increased (Imtiaz et al., 2017). A topical lotion containing *S. repens* used daily on the scalp of 50 men was shown to treat AGA; a 24-week prospective cohort study found that this product significantly increased overall hair count, especially terminal hair (Wessagowit et al., 2016). In another animal study involving rats, *U. dioica* juice gel was shown to accelerate wool growth, enhance the number of hair follicles in the active anagen phase, and improve wool quality by increasing the weight and reducing the proportion of dystrophic hair (Ivanivna et al., 2018).

Despite their widespread use, most hair-growth products are extracted from plants and contain low percentages of the effective agents, meaning that users often need to apply large quantities to achieve the desired results. Over the past few decades, progress in structural bioinformatics has opened new avenues for drug development and significantly assisted in the cost-effective identification of potential drug candidates. These computational techniques can also be employed to examine ligand–receptor interactions broadly before experimental evaluations, enabling investigators to select the most beneficial candidates for additional analyses. In our study, we selected specific computational analysis models based on their proven effectiveness in evaluating the binding affinities of phytochemicals to 5-AR. The chosen models include molecular docking and MD simulations. Previous studies, such as those by Salmaso and Moro (2018) and Pan et al. (2018), have successfully employed these models to evaluate ligand–receptor interactions in similar contexts. We carefully selected these models because molecular docking allows us to predict how one molecule interacts with another upon forming a stable complex. Owing to its accuracy and reliability, this method is well-suited for our initial screening of phytochemicals to identify potential 5-AR inhibitors. In particular, Salmaso and Moro (2018) demonstrated the effectiveness of molecular docking in identifying stable complexes between ligands and 5-AR. In addition to molecular docking, MD simulations provide valuable insights into the stabilities of ligand–receptor complexes and ligand-encapsulated LNPs. This method allows tracking of molecular behaviors over time to verify the efficacies and stabilities of the encapsulated ligands in a dynamic environment. Our approach builds on the methodology used by Pan et al. (2018), who utilized MD simulations to assess the dynamic behaviors of ligand–receptor complexes. Based on comparisons with other models, these methods are found to provide the optimal balance of accuracy, computational efficiency, and relevance to our research goals (Mani et al., 2023), where we aimed to assess the efficacies of bioactive compounds derived from traditional herbal sources in inhibiting androgen receptors through computational methods.

We utilized small-molecular docking to specify the optimal binding configurations of the selected ligands to 5-AR and predict their binding affinities. The docking results revealed that 16 molecules could bind to the receptor more effectively than other candidates. Among these, quercetin and various kaempferol analogs exhibited strong binding to 5-AR, making them promising candidates for further analyses. Previously, researchers have developed a nanocomposite microneedle containing zinc, copper, and quercetin (ZCQ) and showed that the microneedles dissolve to release the metals and quercetin after injection. Thus, quercetin along with these metals was shown to synergistically promote hair follicle regeneration (Zhang et al., 2023). The authors suggested that this regenerative mechanism may be linked to the suppression of DHT. Our study shows that kaempferol analogs possess acceptable affinities to the 5-AR binding site. In a study conducted by Park et al. (2006), the researchers validated the actions of kaempferol in inhibiting 5-AR. Furthermore, we observed that rutin (as a glycoside) formed three hydrogen bonds with the 5-AR binding site and exhibited a binding affinity of −9.7 kcal/mol. Giuliani et al. (2015) utilized extracts from *Boehmeria nipononivea* to regulate hair follicle tropism and reported that a composition of rutin and polyunsaturated fatty acids could effectively inhibit 5-AR function.

A thorough analysis of the ADME properties before clinical testing can accelerate drug development. Lipinski’s rules are designed to predict the likelihood of inadequate absorption or penetration of potential medications; this analysis is crucial for avoiding drug failure due to insufficient absorption or penetration (Lipinski, 2004; Nursamsiar et al., 2016; Tinworth and Young, 2020). Among the 16 molecules screened in this study through molecular docking analysis, six compounds demonstrated drug-like properties: jamogenin, neodiosgenin, chlorogenic acid, rutin, riboflavin, and ursolic acid.

Ursolic acid has also been identified in the extract of *Melandrium firmum* (MF), which is known to promote hair growth and exhibits 43.5% inhibition of 5-AR. Additionally, it was shown to induce the anagen phase while increasing the hair density as well as number and size of hair follicles in the studied mice (Huang et al., 2019). Shin et al. (2012) demonstrated that treatment with ursolic acid reduced levels of DHT and decreased prostate weight in rats with BPH. Riboflavin is another compound that has shown potential to interact with 5-AR; a study conducted by Nakayama et al. (1990) indicated the inhibitory activity of riboflavin on 5-AR.

MD simulations have significantly impacted molecular biology and drug development in recent years. The application of MD for studying biological macromolecules has enabled researchers to observe changes in the positions of atoms within proteins and other biomolecules over time (Karplus and Petsko, 1990; Hollingsworth and Dror, 2018). Our MD studies demonstrate that all six screened molecules form stable interactions with the receptor. Chlorogenic acid exhibits robust and stable interactions with the 5-AR enzyme; this compound found in Arabica coffee extract has been shown to promote hair growth by inhibiting 5-AR (Muangsanguan et al., 2023). Additionally, Nguyen et al. (2024) utilized ethyl acetate (EA) to extract flavonoids and physalins from *Physalis angulata* to investigate their effects on BPH via 5-AR inhibition; their analysis indicated that the extract primarily contains rutin and chlorogenic acid.

This study demonstrated that rutin and chlorogenic acid strongly preferred the enzyme, as evidenced by their docking with 5-AR, which yielded binding energies of −9.2 and −9.3 kcal/mol, respectively. In addition to the above effector molecules, neodiosgenin shows good binding affinity and significant interactions with 5-AR. In a recent study by Manju and Devang (2023), an herbal extract formulation containing neodiosgenin and ursolic acid was developed from various traditional medicinal herbs. Among all the MD simulations of the screened ligand–receptor complexes, jamogenin exhibits the most stable interactions with the receptor. Our results indicate that the structural deviation of the receptor when interacting with jamogenin is lower than those of 5-AR forming complexes with the other molecules. Therefore, using MD simulations, we chose jamogenin to compare the stabilities in the encapsulated and free states.

Nanotechnological progression is paving the path for innovative methods of hair follicle regeneration. These advancements include targeted drug delivery systems that increase hair growth and stimulate the activity of gray hair. LNPs are ideal for encapsulating therapeutic agents by rescuing them from degradation and facilitating transport across cell membranes. Our predictions suggest that vesicle-encapsulated jamogenin has greater stability and lower fluctuations than its unencapsulated counterpart. Patzelt and Lademann (2020) developed a nanoparticle emulsion containing 5% minoxidil (MXD-NPs) using a bead mill process; they compared the effects of MXD-NPs with a commercially available minoxidil solution (CA-MXD) on hair growth. The results indicated that MXD-NPs have more pronounced impacts on hair development than CA-MXD. Furthermore, the concentration of minoxidil found in the hair bulge of mice treated with MXD-NPs was 7.4 times greater than that found in mice treated with CA-MXD. Hence, LNP-encapsulated jamogenin may be a promising candidate for further studies to create novel and more effective hair-growth products.

## 5 Conclusion

The present study highlights the potential of six phytochemicals (jamogenin, neodiosgenin, chlorogenic acid, rutin, riboflavin, and ursolic acid) in inhibiting the enzyme 5-AR in the treatment of AGA. Computational analyses revealed that these compounds, particularly jamogenin, have strong binding affinities to 5-AR. Additionally, MD simulations confirmed the stability of jamogenin when encapsulated in LNPs. These findings suggest that the above phytochemicals could be promising candidates for further research and development as natural treatments for hair loss. However, further experimental studies are necessary to assess the functions of these molecules as well as analyze the half-life of LNP-entrapped jamogenin and its effects on hair growth activity.

## 6 Future directions and research recommendations

Future research should prioritize validating the above findings through *in vitro* and *in vivo* studies to prove the efficacy and safety of the suggested compounds in both unencapsulated and encapsulated forms. Additionally, mechanistic studies are suggested for investigating the molecular mechanisms of 5-AR inhibition while optimizing the herbal extraction processes.

## Data Availability

The original contributions presented in the study are included in the article/Supplementary Material, and any further inquiries can be directed to the corresponding author.
